# Influence of polysaccharide‐based edible coatings on enzymatic browning and oxidative senescence of fresh‐cut lettuce

**DOI:** 10.1002/fsn3.2052

**Published:** 2021-01-08

**Authors:** Li Li, Ping Yi, Changbao Li, Ming Xin, Jian Sun, Xuemei He, Jinfeng Sheng, Zhugui Zhou, Fengjin Zheng, Jiemin Li, Guoming Liu, Dongning Ling, Jie Tang, Zhichun Li, Ying Yang, Yayuan Tang

**Affiliations:** ^1^ Agro‐food Science and Technology Research Institute Guangxi Academy of Agricultural Sciences Nanning China; ^2^ Guangxi Key Laboratory of Fruits and Vegetables Storage‐processing Technology Nanning China

**Keywords:** alginate, carrageenan, chitosan, color, enzymatic browning, fresh‐cut lettuce, oxidative senescence, polysaccharide‐based edible coatings, shelf‐life

## Abstract

Fresh‐cut lettuce has a short shelf‐life due to enzymatic browning and oxidative senescence. The present study investigated effects of polysaccharide‐based edible coatings (alginate, chitosan, and carrageenan) on enzymatic browning and antioxidant defense system of fresh‐cut lettuces during cold storage (4°C) for 15 days. The results showed that three coatings could inhibit enzymatic browning through maintaining total phenolics (TP) content and decreasing polyphenol oxidase (PPO) and phenylalanine ammonialyase (PAL) activities. These coatings also reduced phospholipase D (PLD) and lipoxygenase (LOX) activities, lowered malondialdehyde (MDA) content, and enhanced antioxidant enzymes (superoxide dismutase, SOD; peroxidase, POD; catalase, CAT; ascorbate peroxidase, APX) activities. Besides, all coatings positively affected sensory properties of fresh‐cut lettuces after 3 days storage. Additionally, among three coating treatments, chitosan coating had the most positive effects on quality of fresh‐cut lettuce and was the most suitable coating for retarding enzymatic browning and alleviating membrane oxidative damage. These results indicated that polysaccharide‐based edible coatings were helpful to maintain quality, inhibit enzymatic browning, and postpone senescence of fresh‐cut lettuce.

## Graphical abstract

The present study investigated effects of polysaccharide‐based edible coatings (alginate, chitosan, and carrageenan) on enzymatic browning and antioxidant defense system of fresh‐cut lettuces during cold storage. The study results will be helpful to select appropriate natural edible coatings for extending shelf‐life and maintain quality of fresh‐cut lettuces.

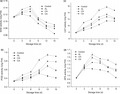

## INTRODUCTION

In recent decades, fresh‐cut vegetable industry has expanded rapidly due to consumers’ demand for fresh, convenient, and healthy foods (Ansah et al., 2018; Martínez‐Hernández et al., 2013). However, the shelf‐life of fresh‐cut products is greatly shortened because of special operations such as washing, peeling, and cutting (Chiumarelli & Hubinger, 2012). Commonly, mechanical wounding causes physiological and metabolic changes, which includes microbial growth, tissue browning, respiration rate increasing, ethylene production, and quality deterioration of fresh‐cut products (Oms‐Oliu et al., 2008). Fresh‐cut lettuce is one of commodities with higher request by salad bars and fast food services, and its market share is increasing constantly (Teng et al., 2019). Fresh‐cut lettuce is susceptible to tissue browning and quality deterioration during storage, even if it is kept in low temperature (Charles et al., 2018). Therefore, methods of effective and safe treatments need to be constantly investigated for alleviating and overcoming these problems.

Membrane deterioration is an early and characteristic feature for the senescence of fresh‐cut vegetable (Dek et al., 2018). The loss of membrane integrity in senescing tissues is caused by the increased lipid peroxidation level, which is mediated by phospholipid‐degrading enzymes, such as phospholipase D (PLD) and lipoxygenase (LOX) (Song et al., 2014). In addition, plant cells possess very efficient enzymatic and nonenzymatic antioxidant defense systems. In the enzymatic antioxidant defense system, a series of antioxidases including superoxide dismutase (SOD), peroxidase (POD), catalase (CAT), and ascorbate peroxidase (APX) plays a very important role in inhibiting oxidative stress in cells (Sun et al., 2011). Hence, phospholipid‐degrading enzymes and antioxidases make valuable synergistic effects to alleviate membrane lipid peroxidation and protect cellular membrane from radical oxygen species (ROS).

The development of edible coatings and films as active ingredient carriers is regarded as a promising packaging strategy to maintain freshness of fresh‐cut fruit and vegetable. Edible coatings can prolong shelf‐life of fresh‐cut fruits and vegetables through reducing moisture, respiration, gas exchange, and rates of oxidative reactions (Senna et al., 2014). It is expected that edible coatings can generate modified atmosphere on the surface of vegetables under optimal relative humidity and refrigeration temperature. The application of polysaccharide‐based edible coatings, which are enriched with antimicrobials or antioxidants, has been confirmed to have great potential for developing high‐quality and long shelf‐life fresh‐cut commodities. For example, alginate, chitosan, and carrageenan are often used as polysaccharide‐based edible coatings because of their ability to form rigid and stable gels (Li et al., 2017). Moreira et al. (2015) reported that alginate coating with dietary fiber addition was helpful for maintaining ideal quality of fresh‐cut apples. Salinas‐Roca et al. (2018) found that chitosan was the most suitable coating to extend fresh‐cut mango quality throughout storage. In addition, Rojas‐Graü et al. (2007) showed that a mixture of chitosan, carrageenan, and sodium alginate inhibited polyphenol oxidase (PPO) activity, enhanced total antioxidant capability, prevented polyphenol oxidation, and reduced browning degree of fresh‐cut yam. Thus, it is suggested that the role of polysaccharide coatings in the senescence of fresh‐cut vegetables may be related to membrane integrity and antioxidant systems. However, little information is available on quality deterioration of fresh‐cut lettuce involves in antioxidant systems and it is important to note that the influence of polysaccharide coatings on antioxidant enzyme system and oxidative senescence of fresh‐cut lettuce has not been studied up to date.

The main objective of our study was to investigate effects of three polysaccharide‐based edible coatings (alginate, chitosan, and carrageenan) on enzymatic browning and oxidative senescence of fresh‐cut lettuce during cold storage. The changes in membrane permeability, lipid peroxidation, lipid‐hydrolyzed enzymes, and antioxidant enzymes were analyzed thoroughly. In addition, the sensory qualities of these polysaccharide‐coated fresh‐cut lettuces were evaluated. The results of this study will be helpful for the fresh‐cut vegetable industry to select appropriate natural edible coatings to extend shelf‐life and maintain quality of lettuce.

## MATERIALS AND METHODS

### Plant materials and treatments

Fresh lettuces (*Lactuca sativa* L. var. *ramosa* Hort.) were harvested from a local farm in Nanning, Guangxi province of China in June 2018. They were immediately transported to a near distributor and kept in a low temperature (4°C) condition with an air‐cooling system. After cooling, lettuces were taken to Guangxi Key Laboratory of Fruits and Vegetables Storage‐processing Technology by commercial refrigerated truck (4°C) and stored for 24 hr at 4°C before processing. Then, lettuces have similar sizes and did not show any decay or mechanical damage were chosen as experimental materials. Lettuce leaves were removed manually, washed with tap water, and cut into 3‐mm shreds. Before treatments, these shreds were allocated to 4 different groups in a randomized manner. To make the polysaccharide‐based coating solutions, three polysaccharides (alginate, chitosan, and carrageenan) were placed in sterile water and stirred until they dissolve completely. Above concentrations were selected on the basis of our preliminary study which showed 1.5% alginate, 1% chitosan, and 2.5% carrageenan were optimal coating treatments for retarding fresh‐cut lettuce senescence. Lettuce shreds were dipped in three coating solutions for five minutes. Take lettuce shreds out and make the dry at room temperature for half an hour. Each coated fresh‐cut lettuce (50 g) was packed in polypropylene rigid tray, sealed with polyethylene film and kept at 4°C for a period of 15 days, and measured every 3 days. Uncoated fresh‐cut lettuce was applied as control group which were stored in the same environment as with coated fresh‐cut lettuce.

### Color measurements

The surface color of fresh‐cut lettuce was measured quickly after unfolding packages of coated and uncoated samples on each designed sampling time point during cold storage. A CR‐400 Chroma Meter was used to measure the following values: CIE L* (lightness), a* (redness), and b* (yellowness). The white plate (Y = 94; x = 0.3132; y = 0.3191; standard CIE illuminate; 2° observer) was used as a reference and color values were expressed as L*. L* was the best parameter related to enzymatic browning (Mishra et al., 2012). The Hue angle value representing the color shade was achieved by using the formulation arctan (b*/a*), while the chroma value (C*) which indicates the sample color saturation was achieved by using the formulation (a*^2^ + b*^2^)^1/2^. For each sample, at least 5 reads were taken at different positions of the same lettuce.

### Analysis of enzymatic browning

#### Measurement of total phenolics

The content of total phenolics in fresh‐cut lettuce was measured according to a modified Folin–Ciocalteu method described previously in (Rochín‐Medina et al., 2018). Half gram of grinded fresh‐cut lettuce was placed in a Thermo Forma Orbital shaker and extracted with 2.5 ml extraction buffer containing 3.5 volume of methanol, 3.5 volume of acetone, 3 volume of sterilized water, and 1% formic acid. The mixed sample was isolated by centrifugation at 12,000 g and performed at 4°C temperature for 15 min. After centrifugation, 1,000 μL supernatant containing soluble phenolics was applied with 1,000 μl Folin–Ciocalteu phenol reagent and 2,000 μl 350 mM NaOH. The samples were kept at room temperature for 5 min, and the results were measured by a UV‐160A spectrophotometer at the absorbance of 760 nm. Three measurements were determined to average the readings. Total phenolics content was calculated by milligrams of the standard gallic acid on fresh weight (FW) (mg gallic acid equivalents/FW). Three replicates were carried out in this study.

#### Enzymatic activity assay of PAL and PPO

PAL activity was determined as described previously by Sun et al., (2012). About half gram of grinded fresh‐cut lettuce was homogenized in 2.5 ml sodium borate solution (0.05 M, pH 8.8) containing 2% (w/v) polyvinyl pyrrolidone and 5 mM mercaptoethanol for about 10 min. The step was conducted at 4°C. The mixture was isolated by centrifugation at 12,000g for 15 min. The clear supernatant containing PAL enzyme was collected for further enzymatic assay. The reaction was conducted in a final volume of 3 ml. To start the reaction, 0.1 ml supernatant containing PAL enzyme was applied to 1.9 ml sodium borate solution (0.1 M, pH 8.8) and 1 ml 20 mM l‐phenylalanine. The samples were incubated at 37°C for 1 hr, and the PPO activity was measured by spectrophotometry of absorbance at 290 nm. The reaction was terminated by adding 200 μl 6 M trichloroacetic acid. Each reaction was performed at least three times.

PPO activity was measured according to a modified spectrophotometric method described previously in Sun et al. (2011). In Brief, about half gram of grinded fresh‐cut lettuce was soaked in 2.5 ml Na₃PO₄ solution (0.1 M, pH 6.8) with 0.1% polyvinylpyrrolidone for 10 min. The temperature was kept at 4°C. The mixture was isolated by centrifugation at 12,000g for 15 min. The clear supernatant containing PPO enzyme was applied to 2.9 ml Na₃PO₄ buffer (10 mM, pH 6.8) containing 10 mM catechol, which was used as substrate for the reaction. The samples were incubated at 25°C for 3 min, and the PPO activity was measured by spectrophotometry of absorbance at 400 nm. Three measurements were applied to average the absorbance.

### Analysis of oxidative stress

#### Measurement of malondialdehyde content

Malondialdehyde content was determined and modified according to a previously described method from Sun et al., (2011). Three grams of grinded fresh‐cut lettuce was extracted by 15 ml 10% trichloroacetic acid. The sample mixture was isolated by centrifugation at 15,000 g for 20 min. 1 ml supernatant was taken to mix with 3 ml 0.5% 2‐thiobarbituric acid. Samples were incubated at 95°C for 20 min and then transferred quickly to an ice‐water bath.

After centrifuged at 3,000 g for 10 min, results were measured by spectrophotometry of absorbance at 532 nm, and values read at 600 nm was used as nonspecific backgrounds. At least three measurements were applied to average the absorbance. The malondialdehyde content was calculated using the calculation formula: (μM/g FW) = [6.45 (OD_532_–OD_600_)–0.56 OD_450_] × 5 ml/0.25 g.

#### Enzymatic activity assays of antioxidative‐related enzymes

The activities of CAT, POD, and APX were determined according to two previously described methods of Song et al., (2014) and Peng et al. (2014). At 4°C, three grams of grinded fresh‐cut lettuce was soaked by 0.1 M 2.5 ml Na₃PO₄ solution (0.1 M, pH 7). The mixture was isolated by centrifugation at 12,000 *g* for 15 min. The supernatant was taken for further reactions to determine activities of CAT, POD, and APX. For CAT activity, 200 μL enzyme solution (supernatant) was added to a buffer containing 50 mM Na₃PO₄ pH 7.8 and 15 mM hydrogen peroxide. The reaction was conducted at 25°C for 3 min, and the CAT activity was measured by spectrophotometry of absorbance at 240 nm. One activity unit equals to a 0.001 increase in absorbance per min. For POD activity, 100 μl enzyme solution was added to a buffer containing 100 mM Na₃PO₄ pH 7, 200 μL 0.46% hydrogen peroxide, and 4% guaiacol. The sample mixtures were incubated at 25°C for 3 min, and the POD activity was read by spectrophotometry of absorbance at 470 nm. One activity unit equals to a 0.01 increase in absorbance per min. For APX activity, 100 μl enzyme solution was added to a buffer containing 50 mM Na₃PO₄ pH 7, 1.5 μM ascorbic acid, 0.3 μM hydrogen peroxide, and 0.3 μM ethylene diamine tetra acetic acid. The sample mixtures were incubated at 25°C for 3 min, and the APX activity was read by spectrophotometry of absorbance at 290 nm. One unit of APX activity equals to 1 μmol ascorbic acid reduced per min.

The activity of SOD was determined based on a previously described method of Li et al., (2017). Half gram of grinded fresh‐cut lettuce was placed to a volume of 2.5 ml solution containing 50 mM Na₃PO₄ buffer (pH 7.8) and 0.1% polyvinyl pyrrolidone. The mixture was isolated by centrifugation at 12,000 *g* speed for 20 min at 4°C temperature. The supernatant was collected for determining the activity of SOD, which is based on determining the rate change of photochemical reduction of nitroblue‐tetrazole. For the reaction, 50 μl enzyme solution (supernatant) was added to a buffer with 3 ml final volume, which contains 50 mM Na₃PO₄ pH 7.8, 13 mM methionine, 0.1 mM ethylenediaminetetraacetic acid, 63 μM NBT, and 1.3 μM riboflavin. The sample mixtures were kept at 25°C for 10 min, and the SOD activity was read by spectrophotometry at the absorbance of 560 nm. At the same time, samples contain only 50 mM Na₃PO₄ buffer pH 7.8 was measured as control. One unit of SOD activity equals to a change of 50% inhibition of NBT reduction.

### Analysis of lipid peroxidation

Phospholipase D and lipoxygenase activities as well as protein content were evaluated using 1 g of fresh‐cut lettuce powder as plant material based on methods from Lin et al., (2018) and Natalini et al., (2014). One unit of PLD or LOX activity equals to a 0.1 unit increase of OD per minute at the absorption of 520 nm and 234 nm, respectively. The enzyme activities were calculated on units per gram of fresh weight.

### Sensory analysis

Sensory quality of treated and untreated fresh‐cut lettuce was evaluated based on the method from Moreira et al., (2015) with a few adaptions. The sensory properties and acceptability of lettuce during cold storage were determined by 10 regular lettuce consumers, who were recruited by University of Guangxi (China) to attend the evaluation test. After taking out from the cold storage environment, coated and uncoated fresh‐cut lettuces were evaluated immediately by consumers. Before serving to consumers, sample orders of lettuces were randomized. Panelists were required to fill in a questionnaire, which includes items of sensory characteristics that affected fresh‐cut lettuce marketability, such as taste, visual appearance, color, dehydration, and overall preference. For each sample, panelists were asked to choose a score between zero to five. Five representing extreme like, whereas zero means that they cannot accept or even hate the taste. For each sensory characteristic, final scores were achieved by averaging all scores from panelists. The score threshold of taste acceptance was set at three, below which suggests that the shelf‐life of fresh‐cut lettuce was ended from the view of sensory.

### Statistical analysis

All experiments in this study were conducted in a randomized design with at least three replicates (*n* = 3). All statistics for enzymatic assays and measurement of soluble compounds were performed using SPSS 13.0 statistical software. One‐way ANOVA was applied to compare mean values between treatment and control samples. LSD tests (*p* = .05) were applied to test for significant differences among the means of parameters. The results represented mean ± standard error (*SE*) of three replicated determinations.

## RESULTS

### Color changes of polysaccharide‐coated fresh‐cut lettuces during cold storage

**Figure 1:**
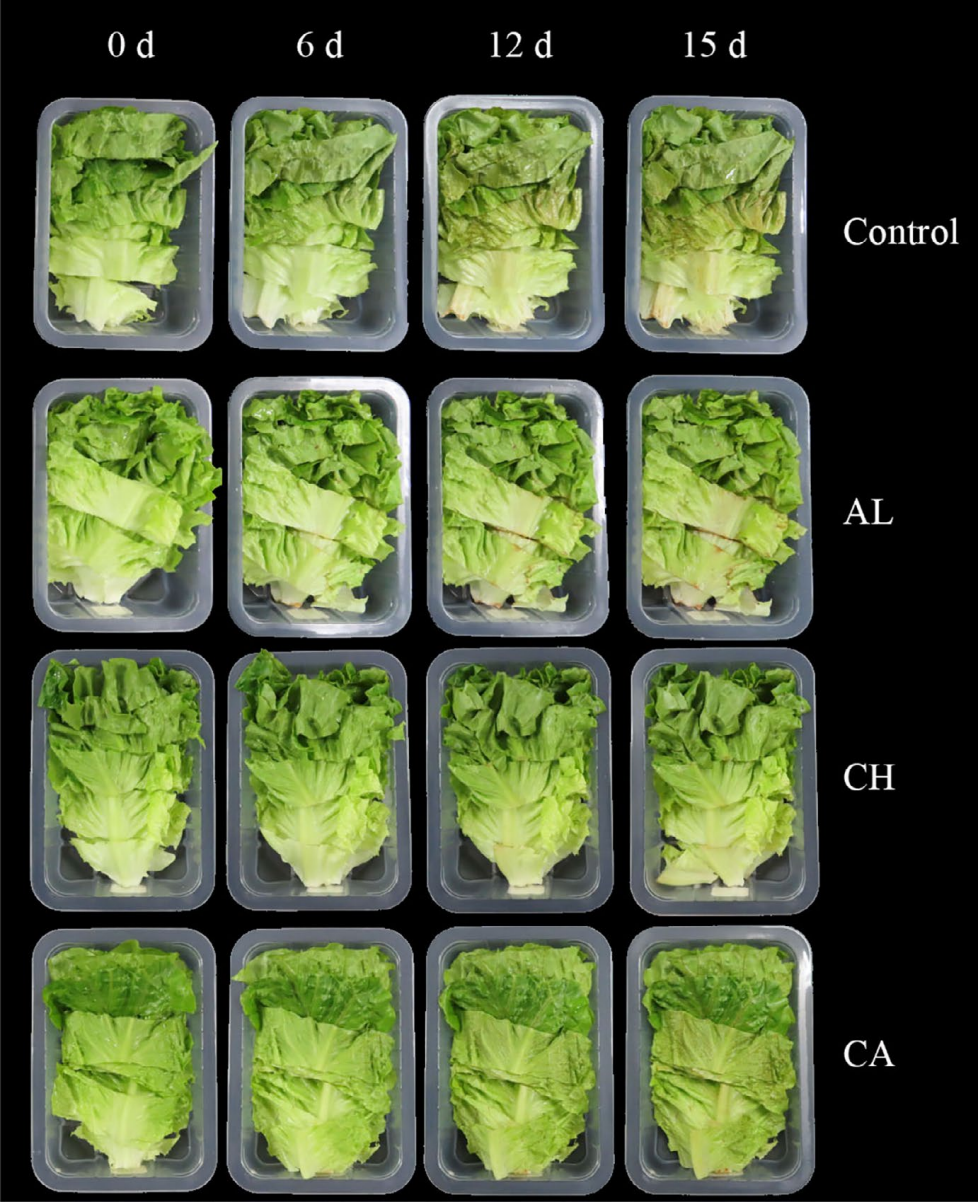
Xxxxxx

**Figure 2:**
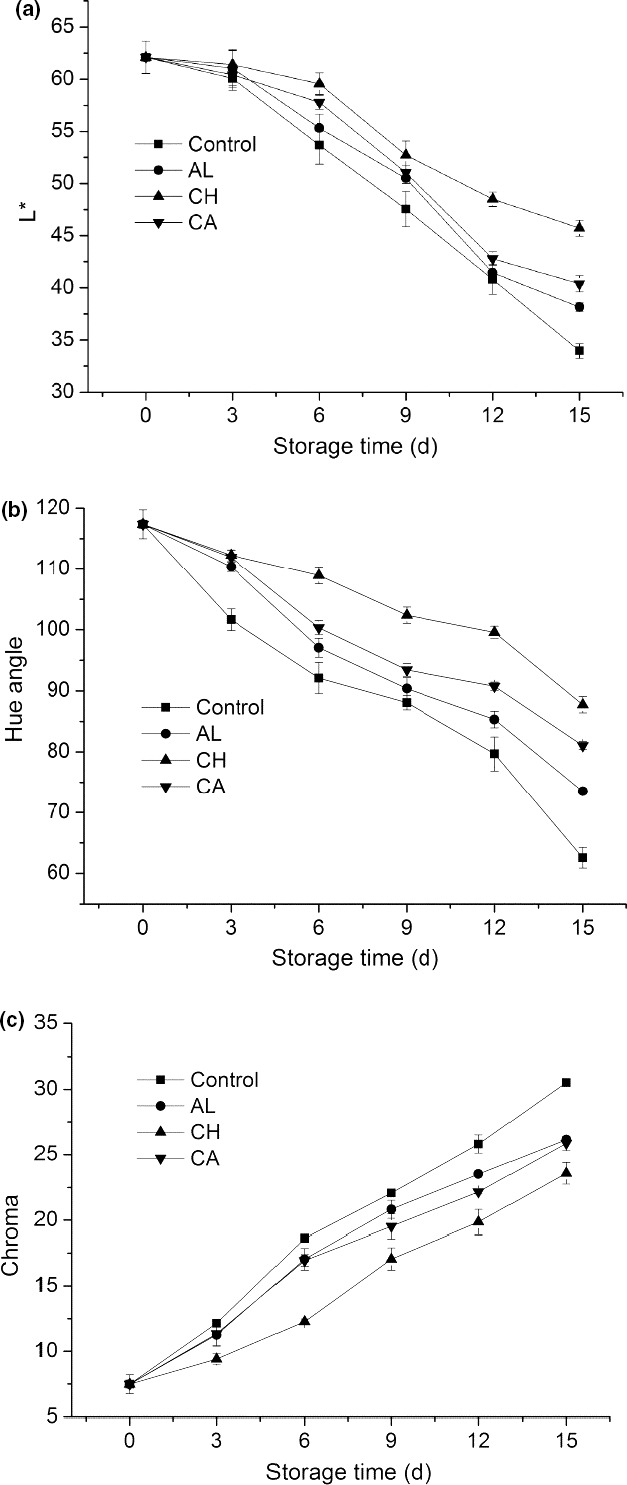
Xxxxxx

Changes in color of fresh‐cut lettuce during storage were also presented in Figure 1. From Figure 1, compared with the control group, three coating treatments (alginate, chitosan, and carrageenan) delayed the surface browning and occurrence of deterioration during storage, and fresh‐cut lettuce treated with chitosan maintained higher overall quality throughout the storage period of 15 days. Three parameters (L* value, hue angle, and chroma) were applied to investigate the color changes of fresh‐cut lettuce. In Figure 2, the obvious decrease of L* value, hue angle, and the significant increase of chroma, indicating that the darkness (browning) was happened and accelerated for all treated lettuce (coated) and control (uncoated) during 15‐d cold storage. Comparing to control, the L* value was delayed (Figure 2a), hue angle was decreased (Figure 2b), and chroma was increased in three groups of polysaccharide‐coated lettuce (Figure 2c). Furthermore, among three coating treatments, chitosan‐coated lettuce exhibited the highest L* value and hue angle as well as the lowest chroma during cold storage. Specifically, from day 6 on, L* value and hue angle of chitosan‐coated lettuce were dramatically larger than those of control (Figure 2a,b), whereas chroma of chitosan‐coated lettuce was significantly lower compared to control (Figure 2c). Therefore, these results suggested that chitosan was the optimal polysaccharide‐based edible coating to effectively inhibit surface browning of fresh‐cut lettuce during cold storage, which can be explained by that chitosan assists to form a thin protective layer on the surface of lettuce to reduce the water transmission.

### Polysaccharide‐based edible coatings delayed enzymatic browning

**Figure 3:**
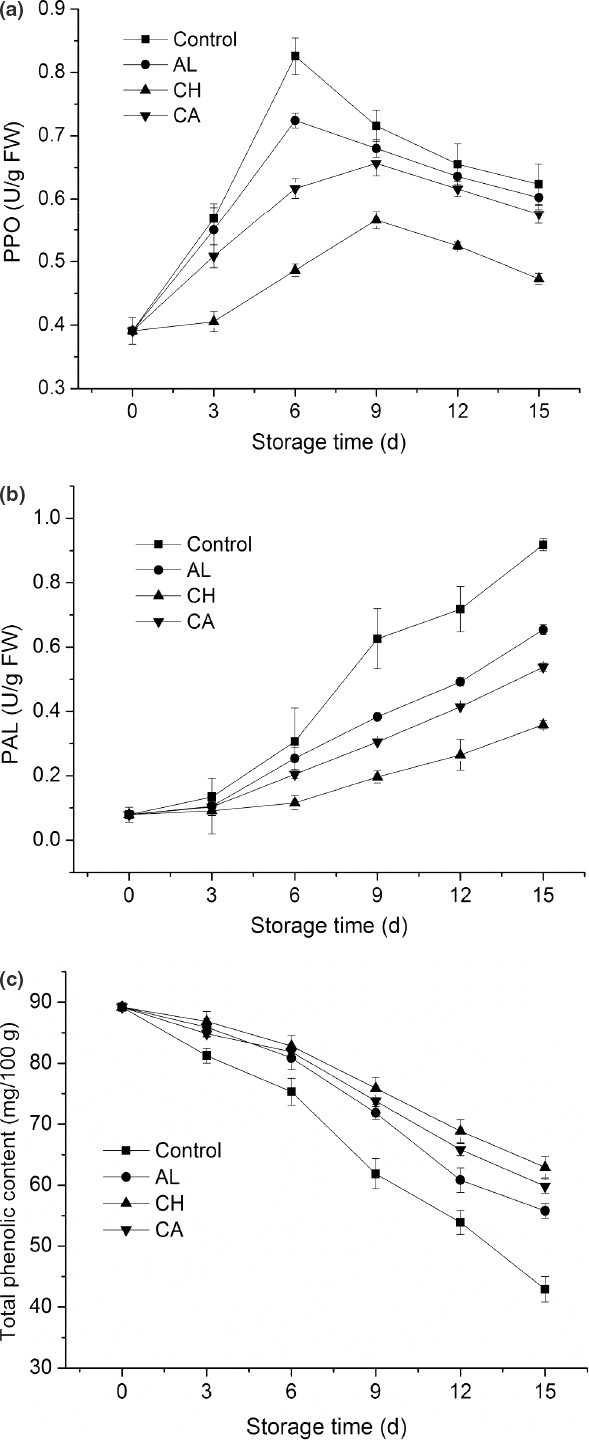
Xxxxxx

PPO plays a key role in enzymatic browning of fresh‐cut vegetables (Mishra et al., 2012). Inhibiting PPO activity is an important method to improve vegetable quality during processing. From Figure 3a, PPO activities in all treatments ascended to peaks in the first a few days and then gradually descended during 15‐d cold storage. Comparing to control, three groups of polysaccharide‐coated lettuce possessed lower PPO activities during storage. In addition, control samples showed the maximum PPO activity on day 6, whereas chitosan and carrageenan‐coated fresh‐cut lettuces presented the maximum PPO activities on day 9. Since both chitosan and carrageenan coatings postponed the time of reaching maximum PPO activity, they were better contributors to inhibit browning of lettuce. Moreover, chitosan‐coated lettuce exhibited the lowest PPO activity among three coating treatments from day 3, indicating that chitosan was the most efficient coating material to inhibit PPO activity of fresh‐cut lettuce.

PAL catalyzes the first reaction in plant defense‐related primary and secondary pathways and plays a key role in producing precursors of a variety of protective phenolic compounds. Phenolics in fresh‐cut lettuce serve as substrates in enzymatic browning reactions. From Figure 3b, three groups of polysaccharide‐coated lettuce had lower PAL activities than control from day 6. Especially, chitosan‐coated lettuce exhibited the lowest PAL activity among three coating treatments from day 6. From Figure 3c, three groups of polysaccharide‐coated fresh‐cut lettuces possessed significantly higher TP contents compared to control during storage. Chitosan‐coated lettuce exhibited the highest TP content among three coating treatments from day 9. Taken together, these results showed that chitosan was the optimum polysaccharide‐based edible coating for inhibiting PPO and PAL activities, reducing phenolic metabolism, and maintaining high TP content so as to retard browning of fresh‐cut lettuce.

### Polysaccharide‐based edible coatings inhibited lipid peroxidation

**Figure 4:**
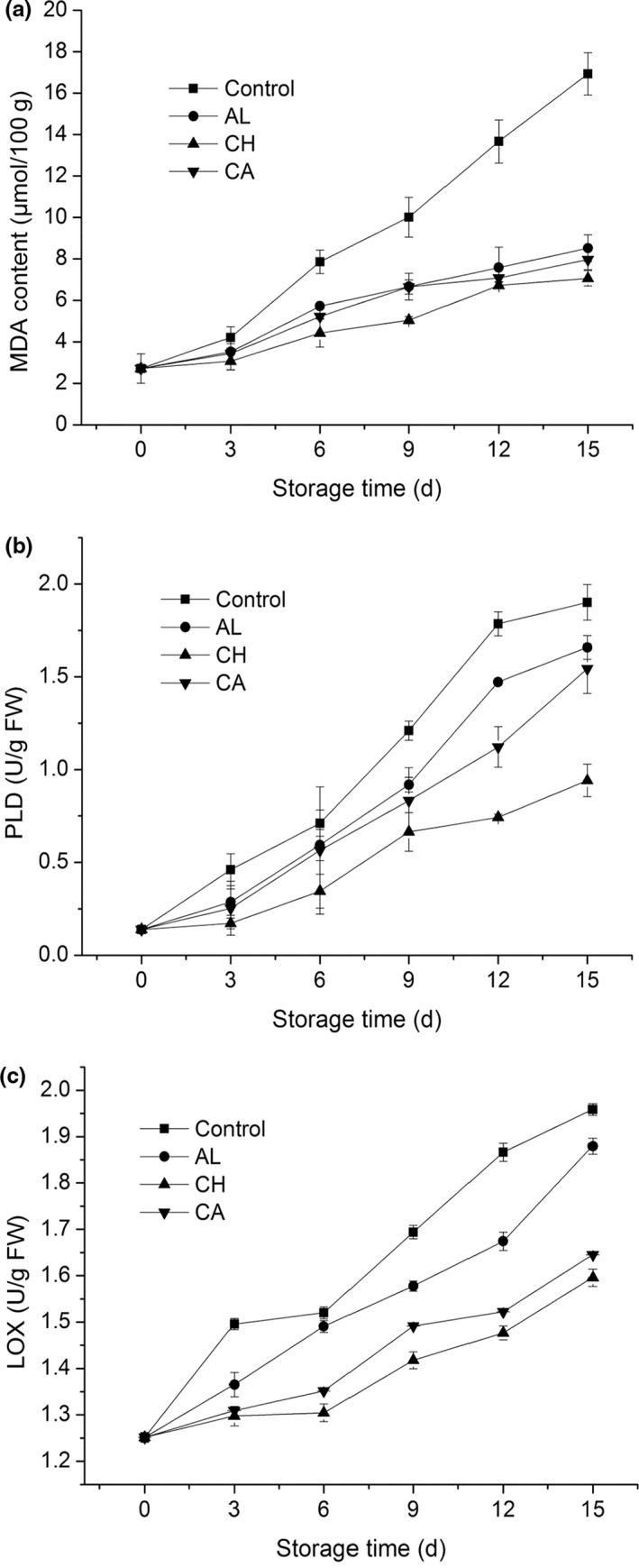
Xxxxxx

MDA is a compound produced in lipid peroxidation of the cell membrane due to ROS accumulation. The change of MDA content can be used as a marker which indicates levels of the lipid peroxidation of membranes in plants, when they are suffering from senescence or stress conditions. From Figure 4a, a steady growth in MDA content was shown for all three edible coating treatments and control during the 15‐d cold storage. Three polysaccharide‐based edible coatings significantly reduced the growth rate of MDA content after day 3 comparing to control. Among three coating treatments, chitosan‐coated lettuce showed the lowest MDA content from day 3. PLD and LOX are crucial participants in phospholipid hydrolysis and membrane lipid peroxidation (Hong et al., 2016; Wang et al., 2019). From Figure 4b, three groups of polysaccharide‐coated fresh‐cut lettuces exhibited relatively lower PLD activity than control during storage. Among three coating treatments, chitosan‐coated lettuce always possessed the lowest PLD activity during the 15‐d storage. From Figure 4c, three polysaccharide coatings, especially chitosan and carrageenan‐coated fresh‐cut lettuce, presented significantly lower LOX activities than control during storage. Moreover, among three coating treatments, chitosan‐coated lettuce showed the lowest LOX activity during storage. Hence, these results indicated that chitosan coating most effectively inhibited the increase of MDA content, as well as reduced PLD and LOX activities in cells of fresh‐cut lettuce, thereby maintaining their membrane integrity and postponing tissue senescence.

### Polysaccharide‐based edible coatings reduced oxidative stress

**Figure 5:**
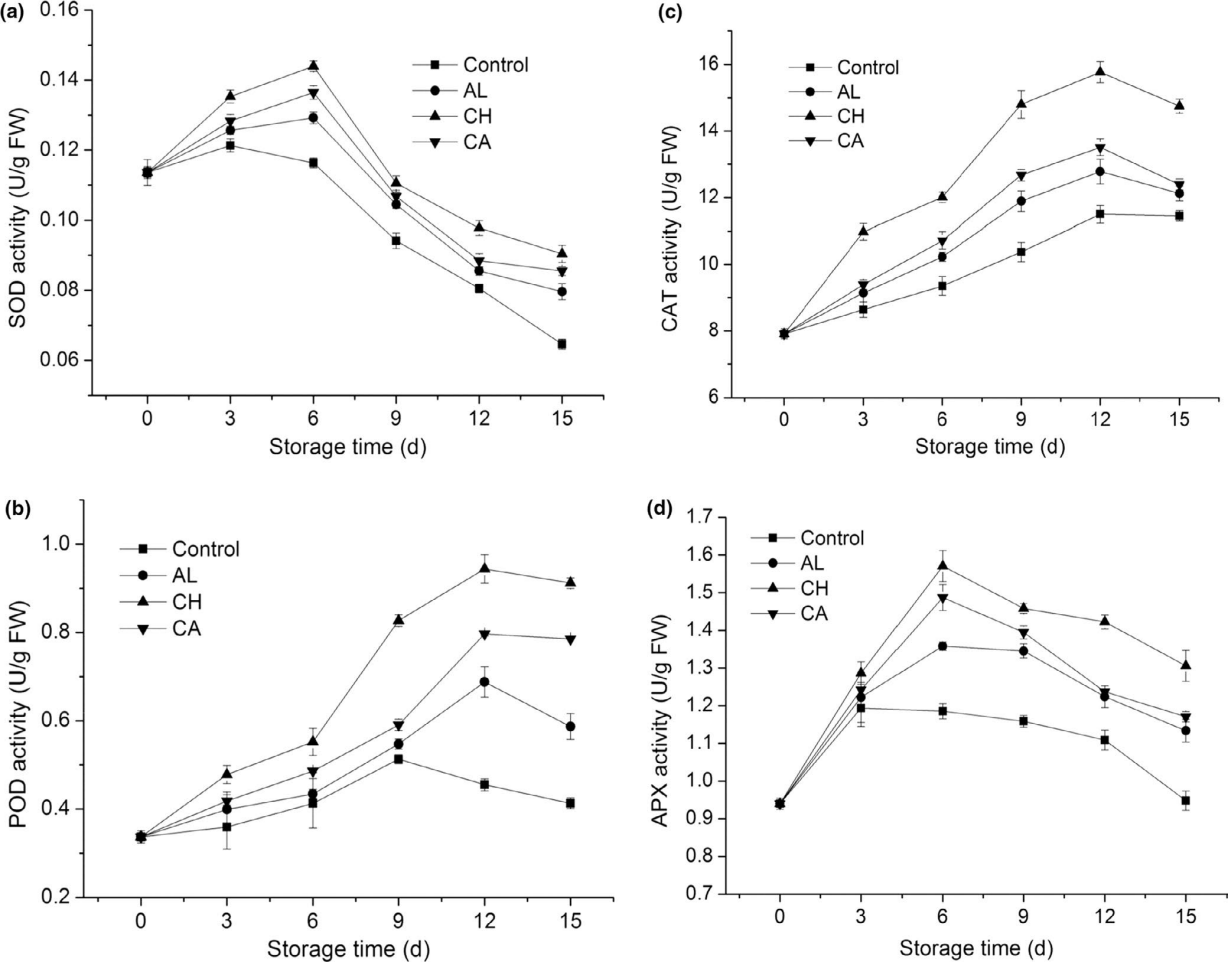
Xxxxxx

Senescence retardation is associated with activities of antioxidant enzymes including SOD, POD, CAT, and APX in a variety of vegetables. From Figure 5a, SOD activities gradually increased in the first a few days and decreased after day 3 in control, whereas the SOD activities just start to decrease after day 6 in three groups of polysaccharide‐coated lettuce. In contrast to control, chitosan‐coated lettuce showed significantly higher SOD activity during 15‐day storage. Moreover, among three coating treatments, chitosan‐coated lettuce displayed the highest SOD activity during storage. POD is a plant defense‐related enzyme and has an essential role in resisting membrane damage mainly through enzymatic degradation of H_2_O_2_. From Figure 5b, POD activities gradually increased and then decreased after day 12 in three groups of polysaccharide‐coated lettuce, whereas the POD activity already start to decrease after day 9 in control. Chitosan‐coated lettuce exhibited significantly higher POD activity compared to control during storage. Furthermore, it exhibited the highest POD activity among three coating treatments during storage. From Figure 5c, CAT activities in all treatments continually increased at the beginning and then decreased after day 12. Three groups of polysaccharide‐coated lettuce possessed higher CAT activities than control during storage. Especially, CAT activity in chitosan‐coated lettuce was the highest among all treatments. From Figure 5d, APX activities rapidly increased and then decreased slowly from day 3 in control samples, whereas it began to decrease from day 6 in three groups of coated fresh‐cut lettuce. These three coated lettuces had significantly higher APX activities than control after day 3. Among them, chitosan‐coated lettuce exhibited the highest APX activity during storage. In summary, chitosan coating could effectively relieve the damage of fresh‐cut lettuce under oxidative stress during storage, by increasing activities of antioxidant‐related enzymes, suggesting that chitosan was an optimal polysaccharide‐based coating in quality maintenance of fresh‐cut lettuce.

### Evaluation of sensory acceptability

**Figure 6:**
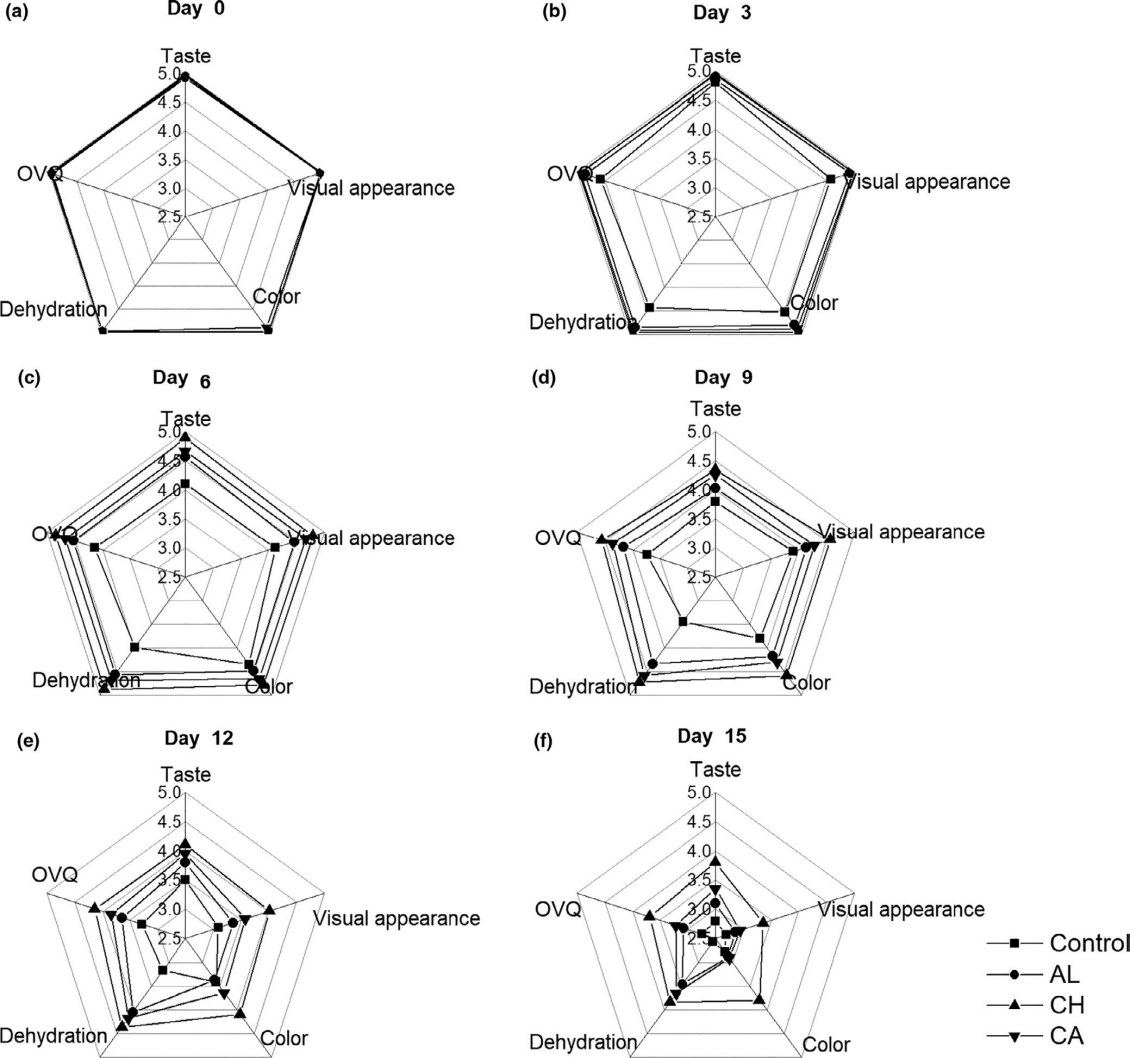
Xxxxxx

Sensory properties including taste, visual appearance, color, dehydration, and overall preference (OVQ) were evaluated among three groups of polysaccharide‐coated fresh‐cut lettuce and control. From Figure 6, lettuce with three coating treatments initially obtained higher scores on visual appearances, color, and OVQ than control, whereas no differences were displayed in dehydration scores between treated and control samples. Alginate and carrageenan‐coated fresh‐cut lettuce gained lower taste scores comparing to both chitosan‐coated lettuce and control samples on day 3. Generally, sensory scores of all samples gradually decreased with the extended storage time. However, three coatings enhanced sensory properties of lettuce after day 9. Among three coating treatments, chitosan‐coated lettuce showed the highest scores on taste, visual appearance, color, dehydration, and OVQ. According to a previous report, fresh‐cut lettuce with sensory scores below 3 was defined as unmarketable (Moreira et al., 2015). After day 15, the scores on all sensorial attributes were above sensory acceptability for chitosan‐coated lettuce, indicating that chitosan treatment was well accepted by panelists.

## DISCUSSION

Active edible coating is a recently developed promising food packaging technique to extend shelf‐life of agricultural products, through providing a semipermeable barrier to gases and water vapor, and reducing enzymatic browning, water loss, and rate of respiration (Badawy et al., 2017). Polysaccharides such as chitosan, alginate, carrageenan, gellan, and starch have been reported as safe natural biodegradable materials to prepare edible coatings (Azarakhsh et al., 2014). Several polysaccharide‐based edible coatings have been used to effectively extend the storage time of fruits and vegetables, such as melon (Poverenov et al., 2014), potato (Wu & Chen, 2013), carrot (Arjun et al., 2015), cucumber (Fan et al., 2019), and lotus root (Huang et al., 2016). These coatings had remarkable influences on maintaining food quality such as enzymatic browning, loss of texture, and undesirable odors and flavor development.

Color is a key quality attribute of fresh‐cut vegetables, since cutting operation may lead to enzymatic browning, which limits the development of fresh‐cut vegetable industry. In this study, effects of three coatings (chitosan, alginate, and carrageenan) on enzymatic browning and oxidative senescence of fresh‐cut lettuce were investigated. L* value, hue angle, and chroma were used as indicators for enzymatic browning reactions (Figures 1 and 2). Lettuce treated with three coatings showed delayed L* value, reduced hue angle, and enhanced chroma, indicating that these coatings can efficiently keep fresh‐cut lettuce free from browning during 15‐d cold storage. This result was in line with the result in a previous study (Oms‐Oliu et al., 2008). In addition, our research indicated that chitosan‐coated lettuce had the most dramatic effects on color maintenance among all treatments, since chitosan coating could better retain antioxidant properties and inhibit browning of fresh‐cut lettuce.

Enzymatic browning is a widespread color reaction occurring in vegetables, which involves a variety of interactions of oxygen, phenolic compounds, and PPO (Wills, Pristijono, Golding, 2007). PAL is the first committed enzyme in phenylpropanoid metabolism, and the increase of its activity leads to the production of more phenolic compounds which are substrates for PPO (Liu et al., 2014). Kang and Saltveit (2002) found that sustained wounding during fresh‐cut preparation induced PAL expression in lettuce tissue, resulting in the synthesis and accumulation of phenolic compounds, which contributed to the subsequent tissue browning. Inhibition of PPO and PAL activities is an important method to improve the quality of fresh‐cut vegetables during storage. In this study, the results showed that chitosan coating is the most effective approach to inhibit PPO activity and postpone time to reach its maximum level (Figure 3a). Moreover, chitosan coating is also the optimal method that effectively inhibited the decrease of TP content and the increase of PAL activity in fresh‐cut lettuce (Figure 3b,c). Hence, these results mentioned above were consistent with results in previous reports on minimally processed vegetables coated with various carbohydrate polymers (Lee et al., 2003; Moreira et al., 2015; Oms‐Oliu et al., 2008).

Membrane deterioration is an early typical sign for the senescence of fresh‐cut vegetables, leading to enzymatic browning, oxidative stress, and ROS accumulation (Song et al., 2014). The accumulated ROS further results in lipid peroxidation, induces membrane injury, and accelerates cell senescence of vegetables. The peroxidation of membrane lipids produces toxic compounds such as MDA, which is generated in polyunsaturated fatty acid oxidation. In the present study, chitosan‐coated lettuce had the lowest MDA content among all treatments from day 3 (Figure 4a), indicating that chitosan coating most efficiently controlled the overproduction of ROS and suppressed lipid peroxidation after 3‐d storage. A previous report has proved that there was a direct relationship between lipid peroxidation and phospholipid hydrolysis, and phospholipid‐degrading enzymes (such as PLD and LOX) were participated in membrane deterioration and lipid hydrolysis in plants during senescence (Li et al., 2017). In this study, three groups of polysaccharide‐coated fresh‐cut lettuce showed relatively lower PLD and LOX activities than control (Figure 4b,c), indicating these coatings obviously inhibited PLD and LOX activity, thereby maintaining the integrity and stability of membrane, and further postponing tissue senescence. On the other hand, antioxidant enzymes such as SOD, POD, CAT, and APX have an essential role in protecting cells from oxidative damage by scavenging ROS (Azarabadi et al., 2017). In this study, three groups of polysaccharide‐based coatings helped to dramatically maintain high activities of CAT, POD, SOD, and APX in fresh‐cut lettuce during storage (Figure 5). These antioxidant enzymes could prevent excessive production of ROS, delay membrane lipid peroxidation, retard loss of membrane function, and relieve oxidative stress in tissues. Especially, chitosan was the best coating to inhibit PLD and LOX activities as well as maintain antioxidative enzyme activities, indicating that chitosan coating was the optimal approach to retain membrane integrity and suppress oxidative stress, thereby controlling the senescence and decay of fresh‐cut lettuce during storage.

Many edible coatings have bitter or astringent off‐flavors that lead to rejection of products by consumers (Moreira et al., 2015). In this study, three polysaccharide‐based coatings did not cause much difference in color, taste, and OVQ with uncoated lettuce (Figure 6), suggesting that they did not change original sensory attributes of fresh‐cut lettuce. Moreover, three coatings exhibited positive effects on sensory properties, and specifically, chitosan‐coated lettuce was well accepted by panelists after 15‐d storage. This result is in line with a previous study showed that chitosan coating maintained the appearance and texture of fresh‐cut mango (Salinas‐Roca et al., 2018). Similarly, Han et al. (2006) demonstrated that chitosan coating increased appearance and acceptance of strawberries. Therefore, these results can be explained by the fact that, comparing to other polysaccharide‐based coatings, chitosan has an antifungal and film‐forming property, which can form a protective barrier on plants surface to reduce gas exchange of oxygen, inhibit oxidation processes, and maintain sensory properties of plants (Silva et al., 2018).

In summary, three polysaccharide‐based edible coatings, chitosan, alginate, carrageenan, all significantly delayed browning, maintained appearance, and slowed senescence of fresh‐cut lettuce. On the one hand, polysaccharide‐based coatings treatment somewhat inhibited the PPO and PAL activities to reduce phenolic metabolism and decelerate enzymatic browning reactions. On the other hand, polysaccharide‐based coatings delayed browning of fresh‐cut lettuce by raising the activity of ROS scavenging enzymes and inhibiting PLD and LOX activities, which helped to reduce MDA accumulation and maintain membrane integrity. Accordingly, ROS overproduction was lessened, and membrane lipid peroxidation was reduced, degradation of membrane lipids was slowed down, and function and integrity of membranes were maintained. Among these three coatings, chitosan was the most suitable coating to maintain the quality of fresh‐cut lettuce throughout storage. Chitosan is obtained by alkaline N‐deacetylation of chitin, with a linear polysaccharide consisting of (1,4)‐linked 2‐amino‐deoxy‐β‐D‐glucan. Unlike other polysaccharide coating materials, chitosan has ability to resist several fungi and induce defense enzymes such as chitinase and chitosanase, which are associated with induced systemic resistance of fresh‐cut vegetables. Besides, chitosan has higher resistance to oxygen and water than other two polysaccharide‐based coatings. The specific molecular mechanism of polysaccharide‐based coatings in suppressing deterioration‐related gene expressions of fresh‐cut vegetables needs to be investigated in further study.

## CONFLICT OF INTEREST

The authors declared that they have no conflicts of interest.

## ETHICAL APPROVAL

This article does not contain any studies with human participants or animals performed by any of the authors.

## Data Availability

The required data are available in raw and final form with corresponding author.

## References

[fsn32052-bib-0001] Ansah, F. A. , Amodio, M. L. , & Colelli, G. (2018). Quality of fresh‐cut products as affected by harvest and postharvest operations. Journal of the Science of Food and Agriculture, 98(10), 3614–3626. 10.1002/jsfa.8885 29327344

[fsn32052-bib-0002] Arjun, P. , Semwal, D. K. , Semwal, R. B. , Mishra, S. P. , Vijayan, A. B. , & Krishnamoorthy, M. (2015). Quality retention and shelf‐life improvement of fresh‐cut apple, papaya, carrot and cucumber by chitosan‐soy based edible coating. Current Nutrition & Food Science, 11(4), 282–291.

[fsn32052-bib-0003] Azarabadi, S. , Abdollahi, H. , Torabi, M. , Salehi, Z. , & Nasiri, J. (2017). ROS generation, oxidative burst and dynamic expression profiles of ROS‐scavenging enzymes of superoxide dismutase (*SOD*), catalase (*CAT*) and ascorbate peroxidase (*APX*) in response to *Erwinia amylovora* in pear (*Pyrus communis* L). European Journal of Plant Pathology, 147, 279–294. 10.1007/s10658-016-1000-0

[fsn32052-bib-0004] Azarakhsh, N. , Osman, A. , Ghazali, H. M. , Tan, C. P. , & Adzahan, N. M. (2014). Effects of gellan‐based edible coating on the quality of fresh‐cut pineapple during cold storage. Food and Bioprocess Technology, 7, 2144–2151. 10.1007/s11947-014-1261-6

[fsn32052-bib-0005] Badawy, M. E. I. , Rabea, E. I. , El‐Nouby, M. A. M. , Ismail, R. I. A. , & Taktak, N. E. M. (2017). Strawberry shelf life, composition, and enzymes activity in response to edible chitosan coatings. International Journal of Fruit Science, 17(2), 117–136. 10.1080/15538362.2016.1219290

[fsn32052-bib-0006] Charles, F. , Nilprapruck, P. , Roux, D. , & Sallanon, H. (2018). Visible light as a new tool to maintain fresh‐cut lettuce post‐harvest quality. Postharvest Biology and Technology, 135, 51–56. 10.1016/j.postharvbio.2017.08.024

[fsn32052-bib-0007] Chiumarelli, M. , & Hubinger, M. D. (2012). Stability, solubility, mechanical and barrier properties of cassava starch – Carnauba wax edible coatings to preserve fresh‐cut apples. Food Hydrocolloids, 28(1), 59–67. 10.1016/j.foodhyd.2011.12.006

[fsn32052-bib-0008] Dek, M. S. P. , Padmanabhan, P. , Subramanian, J. , & Paliyath, G. (2018). Inhibition of tomato fruit ripening by 1‐MCP, wortmannin and hexanal is associated with a decrease in transcript levels of phospholipase D and other ripening related genes. Postharvest Biology and Technology, 140, 50–59. 10.1016/j.postharvbio.2018.02.009

[fsn32052-bib-0009] Fan, K. , Zhang, M. , Fan, D. , & Jiang, F. (2019). Effect of carbon dots with chitosan coating on microorganisms and storage quality of modified‐atmosphere‐packaged fresh‐cut cucumber. Journal of the Science of Food and Agriculture, 99(13), 6032–6041. 10.1002/jsfa.9879 31226218

[fsn32052-bib-0011] Han, C. , Lederer, C. , McDaniel, M. , & Zhao, Y. (2006). Sensory evaluation of fresh strawberries (*Fragaria ananassa*) coated with chitosan‐based edible coatings. Journal of Food Science, 70(3), 172–S178. 10.1111/j.1365-2621.2005.tb07153.x

[fsn32052-bib-0012] Hong, Y. , Zhao, J. , Guo, L. , Kim, S.‐C. , Deng, X. , Wang, G. , Zhang, G. , Li, M. , & Wang, X. (2016). Plant phospholipases D and C and their diverse functions in stress responses. Progress in Lipid Research, 62, 55–74. 10.1016/j.plipres.2016.01.002 26783886

[fsn32052-bib-0013] Huang, Y. , Sun, Y. , Geng, S. , Zhao, H. , Zhou, Y. , Ju, Q. , & Tu, K. (2016). Effect of konjac glucomannan composite coating on the preservation of fresh‐cut lotus root. Food Science, 37(8), 266‐271. (in Chinese).

[fsn32052-bib-0014] Kang, H.‐M. , & Saltveit, M. E. (2002). Antioxidant capacity of lettuce leaf tissue increases after wounding. Journal of Agricultural and Food Chemistry, 50(26), 7536–7541. 10.1021/jf020721c 12475267

[fsn32052-bib-0015] Lee, J. Y. , Park, H. J. , Lee, C. Y. , & Choi, W. Y. (2003). Extending shelf‐life of minimally processed apples with edible coatings and antibrowning agents. LWT‐Food Science and Technology, 36(3), 323–329. 10.1016/S0023-6438(03)00014-8

[fsn32052-bib-0016] Li, L. I. , He, X. , Sun, J. , Li, C. , Ling, D. , Sheng, J. , Zheng, F. , Liu, G. , Li, J. , Tang, Y. , Yi, P. , Xin, M. , Li, Z. , & Zhou, Z. (2017). Responses of phospholipase D and antioxidant system to mechanical wounding in postharvest banana fruits. Journal of Food Quality, 1, 1–8. 10.1155/2017/8347306

[fsn32052-bib-0017] Lin, Y. , Chen, M. , Lin, H. , Lin, M. , Hung, Y.‐C. , Lin, Y. , Chen, Y. , Wang, H. , & Ritenour, M. A. (2018). *Phomopsis longanae*‐induced pericarp browning and disease development of longan fruit can be alleviated or aggravated by regulation of ATP‐mediated membrane lipid metabolism. Food Chemistry, 269, 644–651. 10.1016/j.foodchem.2018.07.060 30100484

[fsn32052-bib-0018] Liu, Y. , Ge, Y. , Bi, Y. , Li, C. , Deng, H. , Hu, L. , & Dong, B. (2014). Effect of postharvest acibenzolar‐S‐methyl dipping on phenylpropanoid pathway metabolism in muskmelon *(Cucumis melo* L.) fruits. Scientia Horticulturae, 168, 113–119. 10.1016/j.scienta.2014.01.030

[fsn32052-bib-0019] Martínez‐Hernández, G. B. , Artés‐Hernández, F. , Gómez, P. A. , & Artés, F. (2013). Comparative behaviour between kailan‐hybrid and conventional fresh‐cut broccoli throughout shelf‐life. LWT ‐ Food Science and Technology, 50(1), 298–305. 10.1016/j.lwt.2012.05.010

[fsn32052-bib-0020] Mishra, B. B. , Gautam, S. , & Sharma, A. (2012). Browning of fresh‐cut eggplant: Impact of cutting and storage. Postharvest Biology and Technology, 67, 44–51. 10.1016/j.postharvbio.2011.12.009

[fsn32052-bib-0021] Moreira, M. R. , Cassani, L. , Martín‐Belloso, O. , & Soliva‐Fortuny, R. (2015). Effects of polysaccharide‐based edible coatings enriched with dietary fiber on quality attributes of fresh‐cut apples. Journal of Food Science and Technology, 52(12), 7795–7805. 10.1007/s13197-015-1907-z 26604352 PMC4648928

[fsn32052-bib-0022] Natalini, A. , Vanesa, M.‐D. , Ferrante, A. , & Pardossi, A. (2014). Effect of temperature and ripening stages on membrane integrity of fresh‐cut tomatoes. Acta Physiologiae Plantarum, 36, 191–198. 10.1007/s11738-013-1399-2

[fsn32052-bib-0023] Oms‐Oliu, G. , Soliva‐Fortuny, R. , & Martín‐Belloso, O. (2008). Edible coatings with antibrowning agents to maintain sensory quality and antioxidant properties of fresh‐cut pears. Postharvest Biology and Technology, 50(1), 87–94. 10.1016/j.postharvbio.2008.03.005

[fsn32052-bib-0024] Peng, X. , Li, R. , Zou, R. , Chen, J. , Zhang, Q. , Cui, P. , Chen, F. , Fu, Y. U. , Yang, J. , & Xia, X. (2014). Allicin Inhibits Microbial Growth and Oxidative Browning of Fresh‐Cut Lettuce (Lactuca sativa) During Refrigerated Storage. Food and Bioprocess Technology, 7(6), 1597–1605. 10.1007/s11947-013-1154-0

[fsn32052-bib-0025] Poverenov, E. , Cohen, R. , Yefremov, T. , Vinokur, Y. , & Rodov, V. (2014). Effects of polysaccharide‐based edible coatings on fresh‐cut melon quality. Acta Horticulturae, 1015, 145–152. 10.17660/ActaHortic.2014.1015.16

[fsn32052-bib-0026] Rochín‐Medina, J. J. , Ramírez, K. , Rangel‐Peraza, J. , & Bustos‐Terrones, Y. A. (2018). Increase of content and bioactivity of total phenolic compounds from spent coffee grounds through solid state fermentation by *Bacillus clausii* . Journal of Food Science and Technology, 55(3), 915–923. 10.1007/s13197-017-2998-5 29487433 PMC5821647

[fsn32052-bib-0027] Rojas‐Graü, M. A. , Tapia, M. S. , Rodríguez, F. J. , Carmona, A. J. , & Martin‐Belloso, B. O. (2007). Alginate and gellan‐based edible coatings as carriers of antibrowning agents applied on fresh‐cut Fuji apples. Food Hydrocolloids, 21(1), 118–127. 10.1016/j.foodhyd.2006.03.001

[fsn32052-bib-0028] Salinas‐Roca, B. , Guerreiro, A. , Welti‐Chanes, J. , Antunes, M. D. C. , & Martín‐Belloso, O. (2018). Improving quality of fresh‐cut mango using polysaccharide‐based edible coatings. International Journal of Food Science & Technology, 53(11), 938–945. 10.1111/ijfs.13666

[fsn32052-bib-0029] Senna, M. M. H. , Al‐Shamrani, K. M. , & Al‐Arifi, A. S. (2014). Edible coating for shelf‐life extension of fresh banana fruit based on gamma irradiated plasticized poly(vinyl alcohol)/carboxymethyl cellulose/tannin composites. Materials Sciences and Applications, 5, 395–415. 10.4236/msa.2014.56045

[fsn32052-bib-0030] Silva, W. B. , Silva, G. M. C. , Santana, D. B. , Salvador, A. R. , Medeiros, D. B. , Belghith, I. , da Silva, N. M. , Cordeiro, M. H. M. , & Misobutsi, G. P. (2018). Chitosan delays ripening and ROS production in guava (*Psidium guajava* L.) fruit. Food Chemistry, 242, 232–238. 10.1016/j.foodchem.2017.09.052 29037684

[fsn32052-bib-0031] Song, L. , Liu, H. , You, Y. , Sun, J. , Yi, C. , Li, Y. , Jiang, Y. , & Wu, J. (2014). Quality deterioration of cut carnation flowers involves in antioxidant systems and energy status. Scientia Horticulturae, 170, 45–52. 10.1016/j.scienta.2014.02.035

[fsn32052-bib-0032] Sun, J. , Li, C. , Prasad, K. N. , You, X. , Li, L. , Liao, F. , Peng, H. , He, X. , Li, Z. , & Zhang, Y. (2012). Membrane deterioration, enzymatic browning and oxidative stress in fresh fruits of three litchi cultivars during six‐day storage. Scientia Horticulturae, 148, 97–103. 10.1016/j.scienta.2012.09.023

[fsn32052-bib-0033] Sun, J. , You, X. , Li, L. , Peng, H. , Su, W. , Li, C. , He, Q. , & Liao, F. (2011). Effects of a phospholipase D inhibitor on postharvest enzymatic browning and oxidative stress of litchi fruit. Postharvest Biology and Technology, 62(3), 288–294. 10.1016/j.postharvbio.2011.07.001

[fsn32052-bib-0034] Teng, Z. , Luo, Y. , Bornhorst, E. R. , Zhou, B. , Simko, I. , & Trouth, F. (2019). Identification of romaine lettuce (*Lactuca sativa* var. *longifolia*) Cultivars with reduced browning discoloration for fresh‐cut processing. Postharvest Biology and Technology, 156, 110931–110942. 10.1016/j.postharvbio.2019.110931

[fsn32052-bib-0035] Wang, L. , Bokhary, S. U. F. , Xie, B. , Hu, S. , Jin, P. , & Zheng, Y. (2019). Biochemical and molecular effects of glycine betaine treatment on membrane fatty acid metabolism in cold stored peaches. Postharvest Biology and Technology, 154, 58–69. 10.1016/j.postharvbio.2019.04.007

[fsn32052-bib-0037] Wills, R. B. H. , Pristijono, P. , & Golding, J. B. (2007). Browning on the surface of cut lettuce slices inhibited by short term exposure to nitric oxide (NO). Food Chemistry, 107(4), 1387–1392. 10.1016/j.foodchem.2007.09.066

[fsn32052-bib-0036] Wu, S. , & Chen, J. (2013). Using pullulan‐based edible coatings to extend shelf‐life of fresh‐cut ‘Fuji’ apples. International Journal of Biological Macromolecules, 55, 254–257. 10.1016/j.ijbiomac.2013.01.012 23376560

